# Production of bioactive liver-targeting interferon Mu-IFN-CSP by soluble prokaryotic expression

**DOI:** 10.1186/s13568-017-0493-z

**Published:** 2017-10-30

**Authors:** Along Liu, Shuiqing Gui, Lun Zhang, Zhaoxia Chen, Yanan Tang, Mingzhu Xiao, Jie Wang, Wenbin Liu, Xiaobao Jin, Jiayong Zhu, Xuemei Lu

**Affiliations:** 1 School of Basic Courses, Guangdong Pharmaceutical University, 280 Wai Huan Dong Road, Guangzhou Higher Education Mega Center, Guangzhou, 510006 People’s Republic of China; 2 Guangdong Provincial Key Laboratory of Pharmaceutical Bioactive Substances, 280 Wai Huan Dong Road, Guangzhou Higher Education Mega Center, Guangzhou, 510006 People’s Republic of China; 3 Pharmaceutical College, Guangdong Pharmaceutical University, 280 Wai Huan Dong Road, Guangzhou Higher Education Mega Center, Guangzhou, 510006 People’s Republic of China; 4 Intensive Care Unit, Shenzhen Second People’s Hospital, The First Affiliated Hospital of Shenzhen University, Shenzhen, 518031 People’s Republic of China

**Keywords:** Liver-targeting interferon, Amino acid mutant, Preferred codon optimized, *Escherichia coli*, Soluble expression, Expression conditions optimized

## Abstract

A novel liver-targeting interferon (IFN-CSP) was successfully over-expressed in our previous work. The in vitro and in vivo investigation revealed that IFN-CSP has significant anti-hepatitis B virus (HBV) effect and liver-targeting capacity. However, due to the IFN-CSP tends to form inclusion bodies in recombinant *Escherichia coli* (*E. coli*), efficient production of the soluble liver-targeting interferon is a challenge. In view of biomedical application, novel strategies for efficiently expressing liver-targeting interferon and overcoming its poor solubility are necessary and important. In the present study, a modified mu-IFN-CSP was designed base on the amino acid mutant of the native IFN-CSP. Meanwhile, the coding sequence of mu-IFN-CSP was optimized for *E. coli* preferred codon and the induction conditions for expression were optimized by an orthogonal test. After amino acid mutant, codon optimization and induction conditions optimization, the solubility of Mu-IFN-CSP in *E. coli* was up to 98.4%. The structural comparison and molecular dynamic simulation showed that the Mu-IFN-CSP formed three structure changes and were more stable than the native IFN-CSP. Tissue sections binding assays revealed that Mu-IFN-CSP was also able to specific binding to liver. In vitro anti-HBV activity assays showed that the soluble Mu-IFN-CSP has improved anti-HBV effect in HepG2.2.15 cells compared to the native IFN-CSP. The present study reports for the first time that liver-targeting interferon Mu-IFN-CSP can be expressed as soluble form, and also contributes to further support its application as liver-targeting anti-HBV medicine.

## Introduction

Interferon (IFN) is a kind of cytokines with the ability to induce antiviral, immunomodulatory and anti-tumor effects (Degasperi et al. 2017; Kotredes et al. 2017; Vu et al. 2016). IFNs have been applied as therapeutic medicine since first cloning and expressing interferon using DNA recombinant technology (Goeddel et al. 1980). However, some shortcomings such as no organ-specific affinity have limited the therapeutic efficacy of interferon (Suginoshita et al. 2001). Molecular targeted drugs may provide promising strategies for new interferon design (Kato 2015). In our previous study, human IFN α2b have been successfully fused with *Plasmodium* region I peptide to construct a novel liver-targeting fusion interferon (IFN-CSP) (Lu et al. 2014). Use HepG2.2.15 cells and HBV-transgenic mice as in vitro and in vivo model, the investigation revealed that IFN-CSP has significant anti-hepatitis B virus (HBV) effect (Lu et al. 2015a, b). The in vitro and in vivo study verified that the novel liver-targeting interferon specific targeting to liver tissue (Lu et al. 2016; Wang et al. 2015). Thus, the novel liver-targeting interferon may be an excellent substitute for IFN α2b as anti-HBV medicine.

As the interferon genes do not have introns (Radhakrishnan et al. 1996) and *Escherichia coli* (*E. coli*) can grow rapidly, the majority of human interferon has been expressed using recombinant *E. coli* as host cell up to now. IFN α2b contains 165 amino acids, and cysteines at position 1 and 98 as well as 29 and 138 formed two disulfide bonds (Gull et al. 2013). Overexpression of recombinant IFN α2b often result in protein misfolding in *E. coli* cytoplasm, and consequent aggregation into inclusion bodies, which are insoluble and usually biologically inactive (Neves et al. 2004). The downstream protein refolding procedures from inclusion bodies are quite cumbersome and low efficiency (Valente et al. 2004). Since IFN-CSP is a fusion protein combining *plasmodium* region I peptide with human IFN α2b, the problem in the soluble expression of the novel liver-targeting interferon in *E. coli* and the recombinant IFN α2b remains the same. For biomedical application in liver-targeting anti-HBV medicine, efficiently expressing liver-targeting interferon and overcoming its poor solubility are very important.

In the present study, a modified mu-IFN-CSP was designed and synthesized based on the amino acid position mutant of the native IFN-CSP. Meanwhile, the coding sequence of mu-IFN-CSP was optimized for *E. coli* preferred codon and the induction conditions for expression were optimized by an orthogonal test. To clarify the structure–function relationships and investigate the influence of the mutation on the stability of the novel liver-targeting interferon, the three-dimensional structural models of the native IFN-CSP and the mutant Mu-IFN-CSP were constructed and the molecular dynamic simulations were conducted. We also compared the liver tissue binding capacity and in vitro anti-HBV effects of native IFN-CSP and mutant Mu-IFN-CSP.

## Materials and methods

### Plasmids and strains

pMD20-T (Takara, Otsu, Japan) was employed to clone gene and pET-21b (Novagen, Madison, USA) was employed to construct expression plasmid. *E. coli* strain DH5α (Novagen, Madison, USA) was employed for gene manipulation and BL21 (DE3; Novagen, Madison, USA) was employed to construct recombinant expression strain. Recombinant IFN-CSP/pET-21b expression plasmid was obtained from Guangdong Provincial Key Laboratory of Pharmaceutical Bioactive Substances, Guangzhou, People’s Republic of China.

### Design, structural models construction and molecular dynamic simulation

To improve the expression level and the poor solubility of IFN-CSP in recombinant *E. coli*, a new mutant was designed based on the native IFN-CSP using amino acid position mutant. Meanwhile, the coding sequence of mu-IFN-CSP was designed based on the preferred codon usage of *E. coli* (http://www.kazusa.or.jp/codon/). To provide convenient restriction enzyme sites for recombinant plasmid construct, *Nde*I/*Xho*I sites were inserted into the 5′- and 3′-ends of the mu-IFN-CSP gene.

To compare the similarity of protein compositions between the native IFN-CSP and the mu-IFN-CSP, the amino acid composition assay was carried out. To clarify the structure–function relationships and study the influence of the mutation on IFN-CSP stability, the three-dimensional structural models of the native IFN-CSP and the mutant Mu-IFN-CSP were constructed and the molecular dynamic simulations were conducted according to previous study (Bao et al. 2015). The known crystal structure of human IFN α2b (PDB ID: 1RH2), which is closely homologous to IFN-CSP, was used to establish the homologous model. The Swiss-PDB viewer software was used to model the three-dimensional structures of the native IFN-CSP and the mu-IFN-CSP. In order to obtain the optimal conformations, the two protein structure models were chosen as the initial coordinates for molecular dynamic (MD) simulations with AMBER 9.0 software package. During the simulation, the root mean square deviation (RMSD) values of backbone atoms relative to the minimized starting structure were used to evaluate the stability of the simulation. The binding free energies of the two protein systems were also monitored over the course of simulations.

### Construction of mu-IFN-CSP and recombinant plasmids

The improved splicing by overlapping extension polymerase chain reaction (SOE-PCR) was applied to synthetize the mutant gene as described before (Lu et al. 2010, 2015b). 16 oligonucleotides were designed and the nucleotide sequences are presented in Table 1. After SOE-PCR, the synthesized full gene fragments of mu-IFN-CSP were recovered and linked to the pMD20-T vector as described before (Lu et al. 2010, 2015b). The constructed cloning plasmid mu-IFN-CSP/pMD20-T was digested by restriction enzyme and the target-gene fragments were ligated into the cloning site of the pET-21b vector according to the procedures described by the manufacturer (Fig. 4a).

**Table 1:** Nucleotide sequences of oligonucleotides designed for assembly of mu-IFN-CSP

Primers	Nucleotide sequence (from 5′ end to 3′ end)
MuIC-1	GGAATTCCATATG **TGTGATCTGCCTCAGA**
MuIC-2	**GTGCCAGGAGAATCAAGGC**ACGACGGTTACCCAGGCTGTGAG**TCTGAGGCAGATCACA**
MuIC-3	**GCCTTGATTCTCCTGGCAC**AAATGCGTCGTATCTCTCCT**TTCTCCTGCCTGAAGGACC**
MuIC-4	**TATCATCAAACTCCTCCTG**TGGGAATTCAAAGTCATGAC**GGTCCTTCAGGCAGGAGAA**
MuIC-5	**CAGGAGGAGTTTGATGATA**AACAGTTCCAGAAGGCTCAA**GCCATCTCTGTCCTCCATG**
MuIC-6	**CTTTTGTGGTAAAGAGGTT**GAAGATCTGCTGGATCATCT**CATGGAGGACAGAGATGGC**
MuIC-7	**AACCTCTTTACCACAAAAG**ATTCATCTGCTGCTTGGGAT**GAGGACCTCCTTGACAAAT**
MuIC-8	**CTTCCAAGTCATTCAGCTG**CTGGTAGAGTTCGGTGCAGA**ATTTGTCAAGGAGGTCCTC**
MuIC-9	**CAGCTGAATGACTTGGAAG**CCTGTGTGATGCAGGAGGAG**CGTGTGGGAGAAACTCCAC**
MuIC-10	**AGTATTTCTTCACAGCCAA**GATGGAGTCCGCATTCATCA**GTGGAGTTTCTCCCACACG**
MuIC-11	**TTGGCTGTGAAGAAATACT**TCCGTCGTATCACTCTCTAT**CTGACAGAGAAGAAATACA**
MuIC-12	**GCATGATTTCTGCACGGAC**AACCTCCCAGGCACAAGGGC**TGTATTTCTTCTCTGTCAG**
MuIC-13	**GTCCGTGCAGAAATCATGC**GTTCCCTCTCTTTATCAACA**AACTTGCAAGAACGTTTAC**
MuIC-14	**CGTAATTTCTCGTTGTC**TTCCTTACGAC**GTAAACGTTCTTGCAAGTT**
MuIC-15	**GACAACGAGAAATTACG**TAAACCAAAACATAAAAAA**TTAAAGCAACCAGCGGA**
MuIC-16	CCGCTCGAGACCA**TCCGCTGGTTGCTTTAA**

Letters in bold type denote the overlapped parts in Mu-IFN-CSP. Restriction sites are underlined

### Protein expression and solubility optimization

The generated mu-IFN-CSP/pET-21b plasmids were transformed into *E. coli* BL21 (DE3) to test the expression and solubility. The culture medium for the recombinant strains is Luria–Bertani (LB) and the conditions and the process for protein expression are as described before (Lu et al. 2010, 2015b). The induced recombinant strains were disrupted by ultrasonication for 10 min in 50 mM Tris–HCl buffer (30% sucrose, 2.5 mM DTT, 1 mM EDTA, pH 8.0). The strain cell lysate was centrifuged (8000*g*, 4 °C, 30 min) to separate the precipitate and soluble parts. To analyze the expression levels and solubility of recombinant protein, 15% sodium dodecyl sulfate polyacrylamide gel electrophoresis (SDS-PAGE) was used. To characterize the antigenicity of the mu-IFN-CSP, western blot analysis was performed, in which the primary antibody and the second antibody were used as previously described (Lu et al. 2015b).

To improve the expression level and the solubility of mutant in *E. coli*, the induction conditions like induction timing, induction temperature, IPTG concentrations, induction time were optimized by an orthogonal experiment (L(25)(5)(4)). Different conditions of induction include OD_600_ (0.3, 0.6, 0.9, 1.2, 1.5), temperatures (25, 28, 31, 34, 37 °C), IPTG concentrations (0.1, 0.2, 0.4, 0.6, 0.8 mM) and induction times (2, 4, 6, 8, 10 h). Samples (15 μL/lane) were examined by 15% SDS-PAGE and Gel-Pro analyzer Version 4.5 software was used to quantify the percentage fraction of mu-IFN-CSP by densitometry. The solubility level was calculated as: solubility = S′/(S′ + P′), where S′ is the amount of mu-IFN-CSP in the soluble supernatant (S), and P′ is the amount of mu-IFN-CSP in the insoluble precipitation (P).

### Protein purification

Due to its His Tag, the mu-IFN-CSP was purified by affinity chromatography with Hispatch-chelating agarose (GE healthcare, Wisconsin, USA). The soluble supernatant was loaded into the HisTrap affinity column, and the conditions and the process for protein purification are as described before (Lu et al. 2010, 2014). To remove endotoxin, the protein sample was applied to polymyxin B column (Bio-Rad, CA, USA), and the lipopolysaccharides (LPS) content of the sample was detected using chromogenic limulus amoebocyte lysate assay (Associates of Cape Cod, MA, USA). The purity of the purified mu-IFN-CSP was assessed by reverse phase high-performance liquid chromatography (RP-HPLC) as described before (Lu et al. 2014, 2015b).

### Tissue sections preparation and binding assays

For liver tissue binding capacity comparison of native IFN-CSP and mutant Mu-IFN-CSP, the immunofluorescence analyses were performed. Balb/c mice (20–25 g) were purchased from the Center for Experimental Animals of Guangdong Province (Guangzhou, China). All research procedures involving mice were approved by the Guidelines for the Care and Use of Experimental Animals, the Guangdong Pharmaceutical University (SYXK (Yue) 2012-0125) and the Guangdong Pharmaceutical University Animal Care and Use Committee, China. The tissue sections preparation, immunofluorescence experiment and binding assays were performed as described before (Lu et al. 2014).

### In vitro antiviral experiments

The in vitro antiviral activities of native IFN-CSP and mu-IFN-CSP were evaluated using HepG2.2.15 cell as model. The cell culture conditions and the process of in vitro antiviral experiment are as described before (Lu et al. 2014, 2015a). The treating time is 6 days and the concentration of native IFN-CSP and mu-IFN-CSP was the same (at final concentrations of 0.3 nmol/L). The cells were collected for intracellular HBV-DNA and hepatitis B s antigen (HBsAg) expression assays. The culture supernatants were collected for HBV-DNA and HBV antigens assays.

### Measurement of HBsAg, HBeAg and HBV-DNA

The enzyme-linked immunosorbent assay (ELISA) was used to measure HBsAg and hepatitis B e antigen (HBeAg) according to the manufacturer’s instructions of HBsAg and HBeAg diagnostic kits (Shanghai Kehua Biotech Co. Ltd., Shanghai, China). The inhibitory level was calculated as: Inhibitory rate (%) = (1 − OD_IFN-CSP or Mu-IFN-CSP_/OD_control_) × 100%. The real-time fluorescent quantification PCR (FQ-PCR) was used to measure the copies of HBV-DNA according to the manufacturer’s recommendations of HBV-DNA test kit (Da-An Gene Co., Guangzhou, China). The inhibitory level was calculated as: Inhibitory rate (%) = (1 − HBV-DNA copies_IFN-CSP or Mu-IFN-CSP_/HBV-DNA copies_control)_ × 100%.

### Immunofluorescent analysis for HBsAg

HBsAg expression was also analyzed by immunofluorescence analysis as described before (Lu et al. 2015a). The treated HepG2.2.15 cells were seeded on coverslip and the primary antibody and the second antibody were goat polyclonal anti-HBsAg antibody (1:200; Novus Biologicals, CO, USA) and Alexa Fluor Cy3-conjugated donkey anti-goat IgG (1:200; Beyotime, Guangzhou, China), respectively. 4′,6-diamidino-2-phenylindole (DAPI) was used for the cells nuclear staining. Finally, fluorescence microscope (Leica, Heidelberg, Germany) was used for immunofluorescence analyses and images capture.

### Statistical analysis

The values of HBsAg, HBeAg and HBV-DNA were expressed as the mean ± standard error of the mean (SEM). The SPSS (version 13.0 for Windows) statistical software was used for statistical analyses. One-Way Analysis of Variance (ANOVA) was used to analyze the differences between mean values. *P* < 0.05 is considered statistically significant.

## Results

### Design, structural models construction and molecular dynamic simulation

**Fig. 1:**
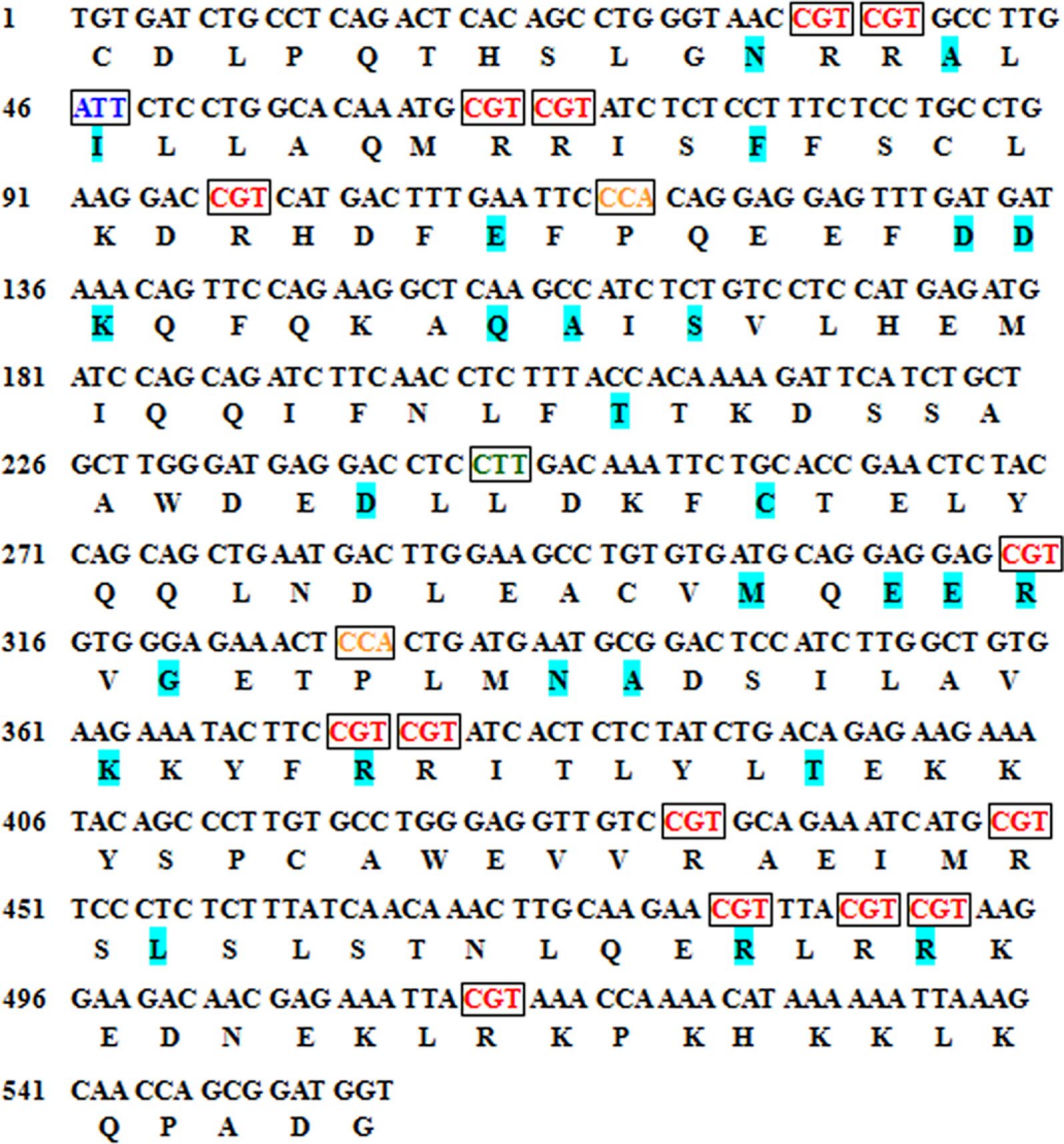
The amino acid sequence and gene sequence of Mu-IFN-CSP. The amino acid residues of Mu-IFN-CSP in the blue shadow are mutation positions. The codons of Mu-IFN-CSP gene in frame are optimized

To obtain a modified liver-targeting interferon with enhanced solubility compared to the native IFN-CSP, we designed a new mutant Mu-IFN-CSP (GenBank Accession No. ALP75560.1) using amino acid position mutant. The mutant contains 185 amino acids, among which 27 amino acids are mutations compared to the native IFN-CSP (Fig. 1). Meanwhile, to eliminate the adverse influence of rare codons on recombinant expression of Mu-IFN-CSP in *E. coli*, the DNA sequence of the mutant gene (GenBank Accession No. KP719982.1) was analyzed and codon-optimized according to the preferred codon usage of *E. coli* (Fig. 1).

**Fig. 2:**
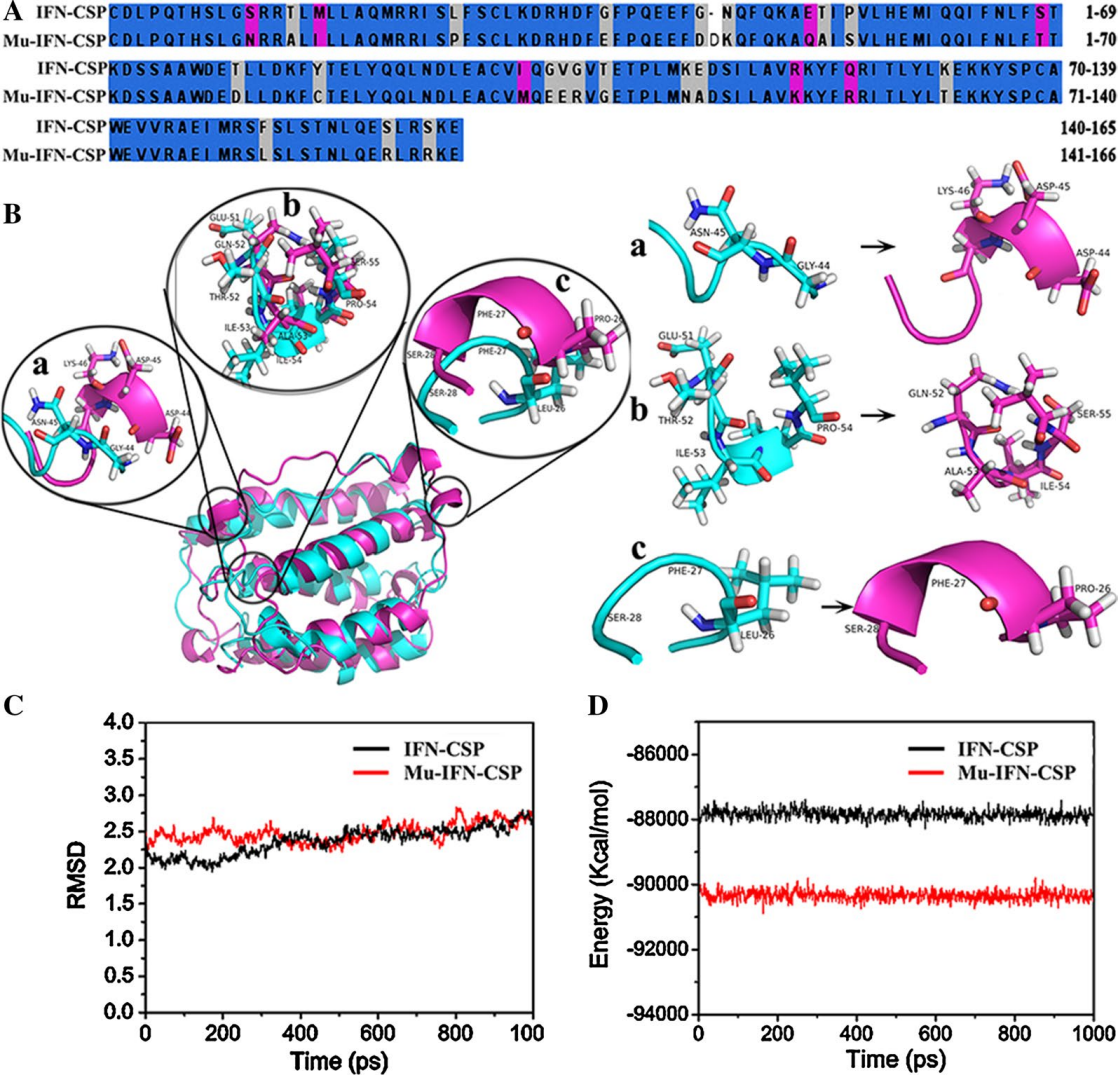
Sequences and structure alignment of native IFN-CSP and Mu-IFN-CSP. **A** Amino acid sequences alignment of native IFN-CSP and Mu-IFN-CSP. The amino acid residues in the blue color regions are identical. The amino acid residues in other color regions are mutation positions. **B** Structure alignment of native IFN-CSP and Mu-IFN-CSP. Green denotes native IFN-CSP; red denotes Mu-IFN-CSP. Compared with native IFN-CSP, the Mu-IFN-CSP formed three structure changes (a, b and c). **C** The RMSDs of the molecular dynamic simulation systems. **D** The total energy of native IFN-CSP and Mu-IFN-CSP during MD simulations

To analyze the key positions of mu-IFN-CSP, the structural models of native IFN-CSP and mu-IFN-CSP were constructed and compared. The comparison result of the amino acid sequences of native protein and mutant was showed in Fig. 2A, where 27 mutation positions were highlighted. Structure alignment of native IFN-CSP and Mu-IFN-CSP was showed in Fig. 2B. Compared with the native protein, the mutant formed three structure changes, there are two random coils turned into alpha helixes (Fig. 2B a, c) and one alpha helixes turned into random coil (Fig. 2B b). The results of RMSDs showed that both the native IFN-CSP and mu-IFN-CSP simulation systems reached equilibrium after 2 ns and the RMSD fluctuations were very small in the remaining 2 ns process (Fig. 2C). The total energy of native IFN-CSP and Mu-IFN-CSP during MD simulations was showed in Fig. 2D. The energy curves of both the native IFN-CSP and mu-IFN-CSP were smooth. The total energy values of the native IFN-CSP and mu-IFN-CSP systems were − 8.8 × 10^4^ and − 9.0 × 10^4^ kcal/mol, respectively.

### Construction of mu-IFN-CSP and recombinant plasmids

**Fig. 3:**
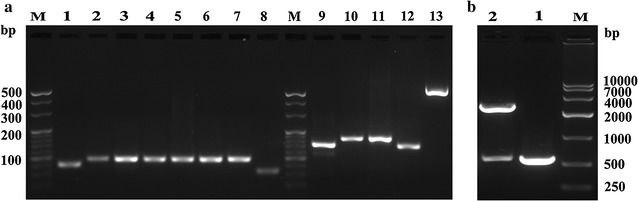
Construction and identification of fusion gene mu-IFN-CSP. **a** Construction of fusion gene mu-IFN-CSP. Lanes 1–8 the first PCR products ranging 66–97 bp in size. Lanes 9–12 the second PCR products ranging 135–175 bp in size. Lane 13 the full-length fusion gene mu-IFN-CSP. Lane M DNA molecular weight marker. **b** Identification of fusion gene mu-IFN-CSP. Lane 1 PCR products of recombinant plasmids. Lane 2 recombinant plasmids mu-IFN-CSP/pMD20-T digested with *Nde*I/*Xho*I. Lane M DNA molecular weight marker

The mu-IFN-CSP gene was constructed by improved SOE-PCR method. The Fig. 3a showed the first and the second PCR products ranging 66–97 and 135–175 bp in size, respectively. The full-length gene of fusion mu-IFN-CSP is 577 bp in size (Fig. 3a Lane 13). The recombinant plasmid mu-IFN-CSP/pMD20-T was identified by PCR screening, DNA sequencing and restriction endonuclease analysis (Fig. 3b). After digested with *Nde*I/*Xho*I, the gene fragment of mu-IFN-CSP was ligated into the cleaved plasmid to construct recombinant mu-IFN-CSP/pET-21b, and recombinant plasmid was verified by the sequencing data.

### Protein expression and solubility optimization

**Fig. 4:**
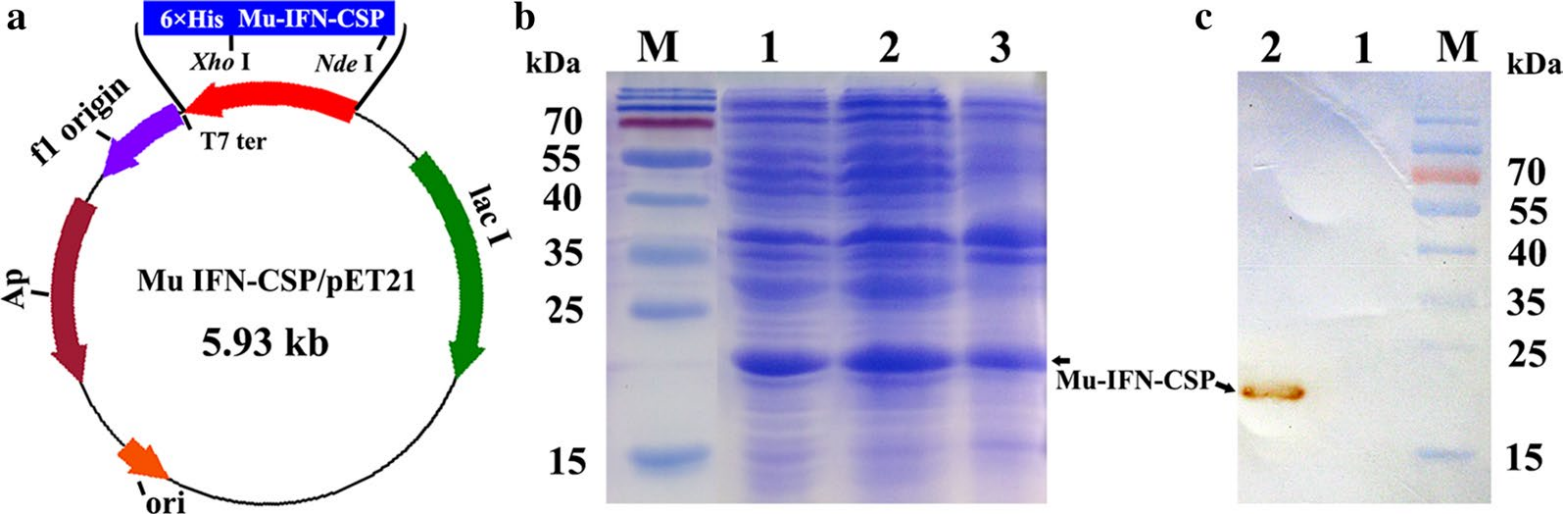
Schematic diagram of mu-IFN-CSP gene in the expression vector mu-IFN-CSP/pET-21b and expression of mu-IFN-CSP protein in recombinant *E. coli*. **a** A schematic diagram of mu-IFN-CSP/pET-21b. **b** SDS-PAGE analysis of mu-IFN-CSP expression. Lane 1 total proteins of recombinant *E. coli* BL21 after IPTG induction. Lanes 2–3 supernatant and precipitation after ultrasonication and centrifugation. Lane M protein molecular weight marker. **c** Recombinant mu-IFN-CSP was analyzed by western blot analysis. Lanes 1–2 total proteins of *E. coli* BL21/pET-21b-mu-IFN-CSP before and after induction. Lane M protein molecular weight marker

The plasmid mu-IFN-CSP/pET-21b was transformed into *E. coli* BL21 (DE3) for protein expression. After induced with IPTG (1.0 mM/L culture) for 4 h, the recombinant protein with 21.5 kD in size was successfully expressed with a 75.2% solubility (Fig. 4b). The result of western blot analysis also shows a specific band in the total protein from recombinant *E. coli* after induction compared with before induction (Fig. 4c).

**Fig. 5:**
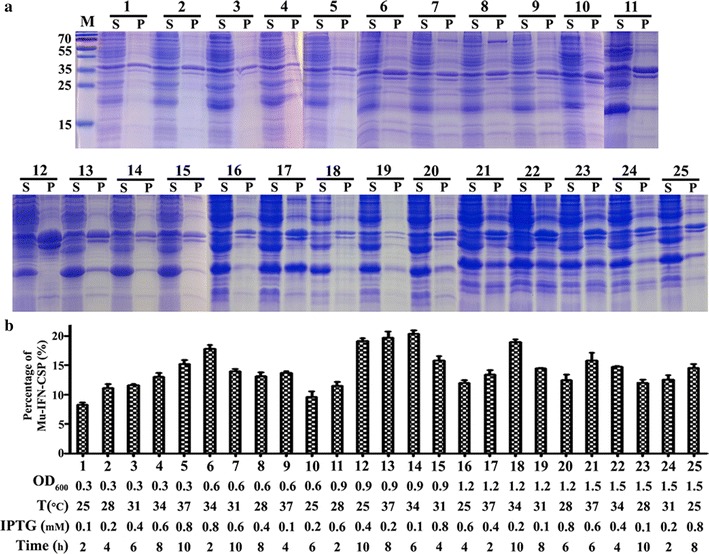
Optimization of mu-IFN-CSP expression by orthogonal test. **a** SDS-PAGE analyses of mu-IFN-CSP expression at different induction conditions. Lane M protein molecular weight marker; *S* soluble supernatant after cell disruption; *P* insoluble precipitation. **b** Percentage of mu-IFN-CSP in the total soluble proteins was calculated by the target bands in SDS-PAGE (**a**)

**Fig. 6:**
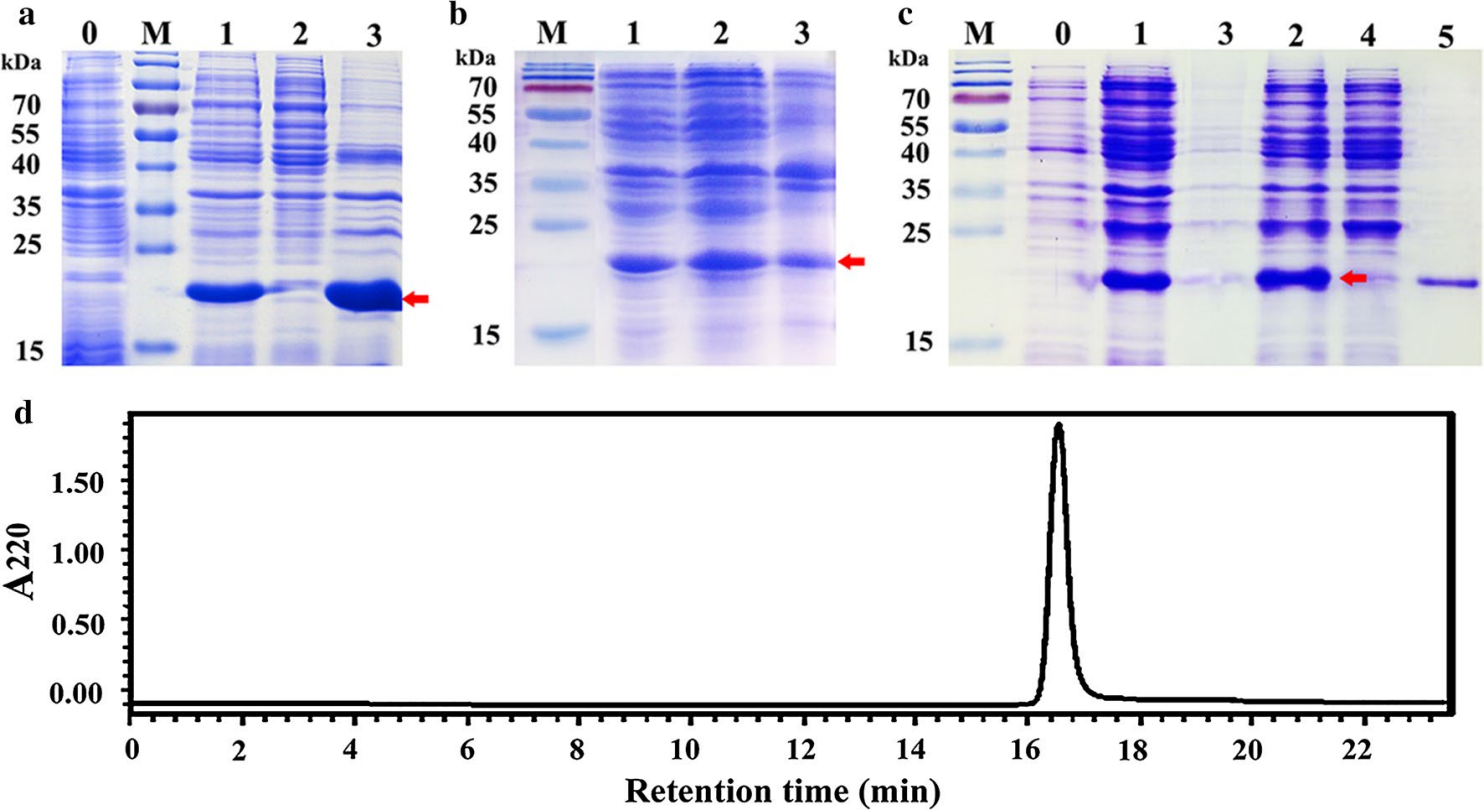
Comparison of the solubility and characterization of recombinant protein. **a** Assay of expression and solubility of IFN-CSP in recombinant *E. coli*. **b** Assay of expression and solubility of mu-IFN-CSP in recombinant *E. coli* before optimization. **c** Assay of expression and solubility of mu-IFN-CSP in recombinant *E. coli* after optimization and purification of mu-IFN-CSP. Lane M protein molecular weight marker. Lanes 0–1 total proteins of recombinant *E. coli* before and after induction. Lanes 2–3 supernatant and precipitation after ultrasonication and centrifugation. Lane 4 eluted protein fraction using HisTrap affinity chromatography. Lane 5 purified mu-IFN-CSP using HisTrap affinity chromatography. The arrows indicate the intense bands at 21.5 kDa corresponding to the target protein. **d** Analysis of purified IFN-CSP by RP-HPLC with a C18 column

Different induction conditions may have great impacts on the solubility and the expression level of recombinant protein in *E. coli*. In present experiment, four parameters including induction timing, induction temperature, IPTG concentrations and induction time were investigated by an orthogonal test. The results showed that the most optimal condition was at OD_600_ = 0.9, inducing the culture at 34 °C for 6 h with 0.1 mM IPTG (Fig. 5). The solubility of Mu-IFN-CSP in *E. coli* was up to 98.4% (Figs. 5b, 6c).

### Protein purification

To obtain mu-IFN-CSP, the soluble supernatant was subjected to a four-step purification process. The first step is affinity chromatography using hispatch-chelating agarose. Figure 6c Lane 4 shows the eluted protein fractions using 100 mM imidazole and Fig. 6c Lane 5 shows the purified mu-IFN-CSP using 500 mM imidazole. The following steps are desalting and endotoxin removing. The eluted product was purified by desalted dialysis and polymyxin B column purification to avoid the negative influence of imidazole and LPS on the bioactivity of mu-IFN-CSP. The forth step is freeze-drying. After polymyxin B column purification, the LPS content in the purified product was less than 0.5 Eu/mg proteins. Figure 6d showed that the RP-HPLC result of mu-IFN-CSP indicating the purity of the protein was over 98%. The yield of the pure soluble mu-IFN-CSP is approximately 64.58 mg/L of *E. coli* culture.

### Tissue sections preparation and binding assays

**Fig. 7:**
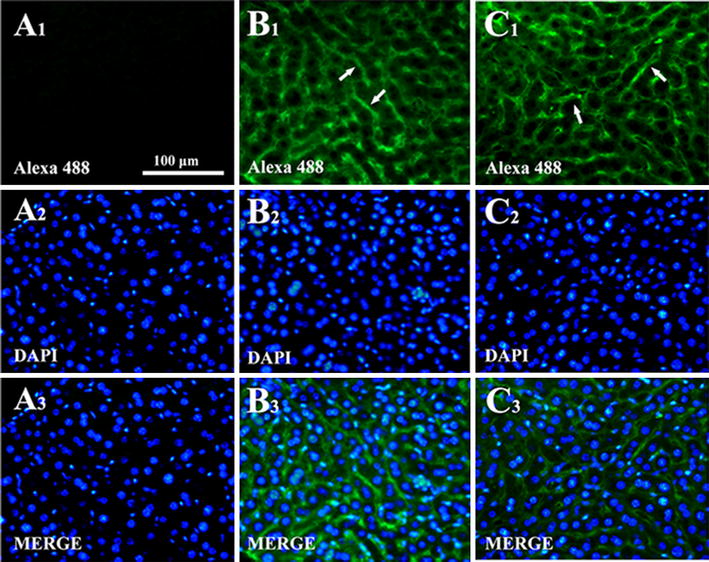
Fluorescence photomicrographs of liver tissue after incubated with IFN-CSP or mu-IFN-CSP. **A** Controls; **B** IFN-CSP; **C** mu-IFN-CSP; **1** green fluorescent labeling of protein stained with anti-IFN antibody; **2** blue nuclear stained with DAPI; **3** merged images of 1 and 2. Arrows indicate examples of distinct green fluorescent labeling of IFN-CSP and mu-IFN-CSP. Calibration bar = 100 μm for all images

To study whether mutant was also able to specific binding to liver, both native protein and mutant were directly applied to liver slices and the immunofluorescence analyses were performed. Figure 7 shows photographs of fluorescent labeling of liver after native IFN-CSP or mu-IFN-CSP incubation. Compared with the control (Fig. 7A), both native IFN-CSP (Fig. 7B) and mu-IFN-CSP (Fig. 7C) treatment displayed green fluorescence, which along the basolateral region and sinusoidal borders.

### Effect of native IFN-CSP and mu-IFN-CSP on HBV antigens secretion

**Fig. 8:**
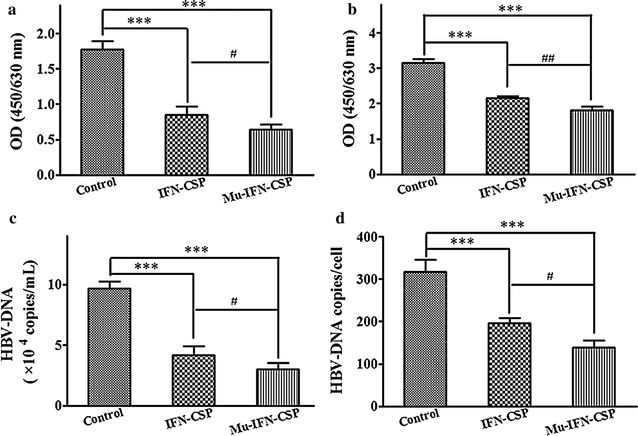
Effect of IFN-CSP or mu-IFN-CSP on HBV antigens secretion and HBV-DNA replication in HepG2.2.15 cells. HepG2.2.15 cells were cultured in the presence of IFN-CSP or mu-IFN-CSP for 6 days. Hepatitis B surface antigen (HBsAg; **a**) and hepatitis B e antigen (HBeAg; **b**) in the culture supernatants were analyzed by enzyme-linked immunosorbant assay (ELISA). Supernatant HBV-DNA (**c**) and intracellular HBV-DNA (**d**) were measured by real-time quantitative PCR. Data represent the mean ± SEM of three experiments. ***P < *0.001* drug group *vs* control group; ^*#*^ *P* < *0.05,* ^*##*^ *P* < *0.01* IFN-CSP group vs mu-IFN-CSP group

The HBsAg and HBeAg in the culture supernatant were measured by ELISA. After treated with native IFN-CSP and mu-IFN-CSP, HBsAg (Fig. 8a) and HBeAg (Fig. 8b) secretion significantly reduced. The inhibition ratios of native IFN-CSP and mu-IFN-CSP on HBsAg were 52.01 and 63.80%, respectively. And the inhibition ratios of native IFN-CSP and mu-IFN-CSP on HBeAg were 31.79 and 42.39%, respectively.

### Effect of native IFN-CSP and mu-IFN-CSP on HBV-DNA

HBV-DNA in culture supernatant and intracellular of HepG2.2.15 cells was measured to further verify the anti-HBV effect of native IFN-CSP and mu-IFN-CSP. The results revealed that culture supernatants (Fig. 8c) and intracellular HBV-DNA (Fig. 8d) of the treated group were significantly decreased. The inhibition ratios of native protein and mutant on HBV-DNA in supernatant were 56.77 and 69.09%, respectively. For intracellular HBV-DNA, the inhibition ratios of native IFN-CSP and mu-IFN-CSP were 37.89 and 56.31%, respectively.

### Effect of native IFN-CSP and mu-IFN-CSP on HBsAg expression

**Fig. 9:**
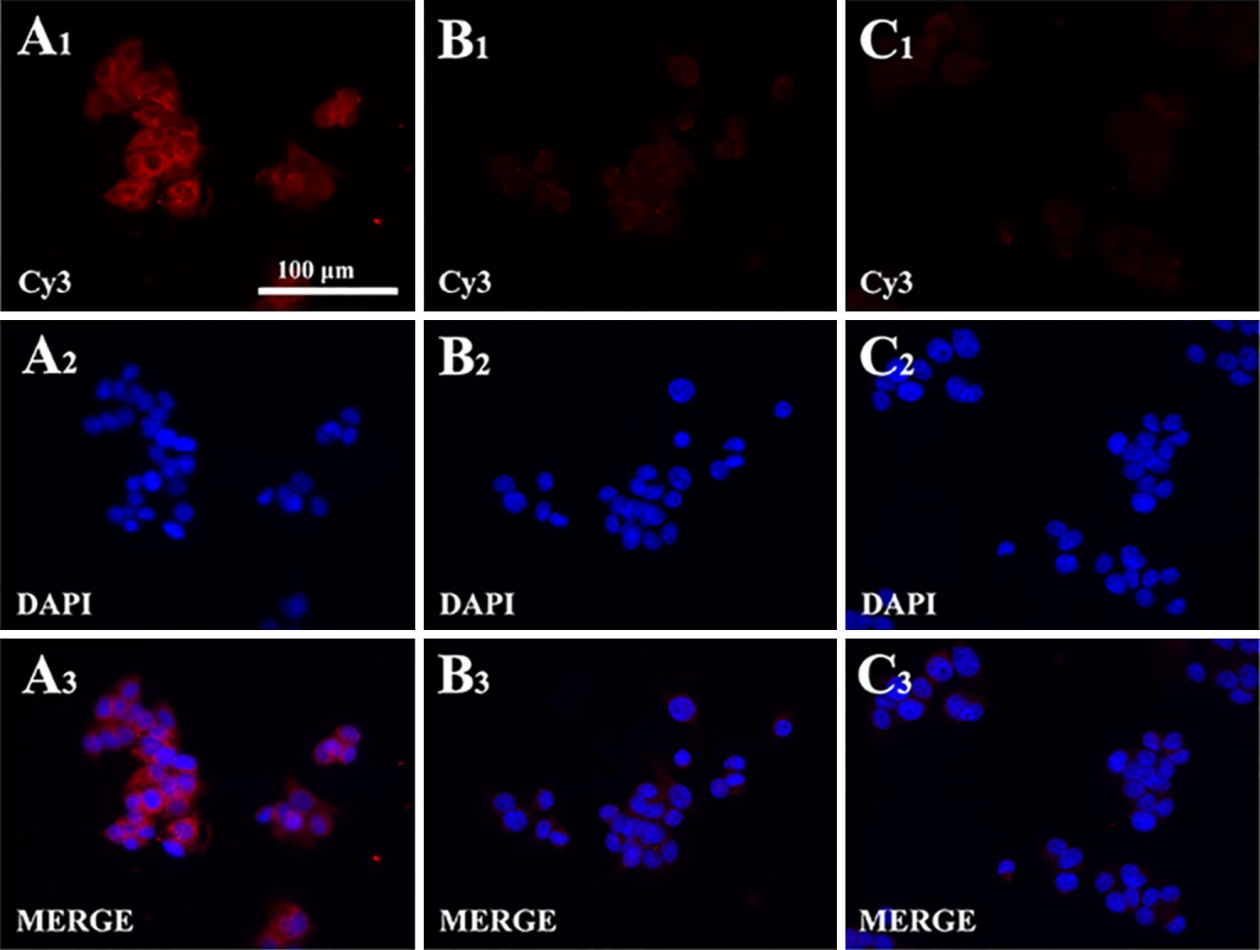
Effect of IFN-CSP or mu-IFN-CSP on HBsAg expression in HepG2.2.15 cells. HepG2.2.15 cells stained by immunofluorescent staining with anti-HBsAg antibody and representative photographs are captured by microscope. **A** Controls; **B** IFN-CSP; **C** mu-IFN-CSP; **1** red stained with HBsAg; **2** blue nuclear stained with DAPI; **3** merged images of 1 and 2. Bar, 100 μm and is the same for all photomicrographs

The immunofluorescence analysis was used to study the HBsAg expression in HepG2.2.15 cells. The fluorescence images captured by microscope showed that the signal of HBsAg is expressed in the cytoplasm and membrane of the HepG2.2.15 cells (Fig. 9). Compared with the control cells, native IFN-CSP and mu-IFN-CSP treated obviously suppress the expression of HBsAg.

## Discussion

The novel liver-targeting interferon IFN-CSP is a promising anti-HBV protein (Lu et al. 2015a, b). However, its efficient production for biomedical applications has been hampered by insoluble inclusion bodies in recombinant *E. coli* expression system and the cumbersome protein refolding process. Intend to express soluble, active recombinant liver-targeting interferon in *E. coli*, several strategies have been used in the expression of natively folded liver-targeting interferon in the present study, including amino acid positions mutation, *E. coli* preferred codon optimize and induction conditions optimize.

At first, based on the native IFN-CSP, we have designed a modified mu-IFN-CSP using amino acid positions mutant. Figure 2A shows the comparison result for the amino acid sequence of the native protein and the mutant, where 27 amino acid positions are mutations. The homology comparison of amino acid sequences of IFN region in the mu-IFN-CSP showed the 100% homology with reported amino acid sequences of human IFN α B/D hybrid (Ghaffar et al. 1992; Horisberger and de Staritzky 1987). The amino acids 1–60 of human IFN α B/D hybrid come from IFN αB and the amino acids 61–166 of IFN α B/D come from IFN αD (Meister et al. 1986). The IFNα B/D hybrid has been found to have comparable or higher antiviral efficacy against vesicular stomatitis virus (VSV), bovine viral diarrhea virus (BVDV) and herpes simplex virus type 1 (HSV-1/VR3) than the parents IFN α2a, IFN αB and IFN αD (Gangemi et al. 1989; Peek et al. 2004). Structure–function investigations of IFN α have found that the differences in activity were correlated with their ability to bind the receptor, and several distinct sequence regions may involve the interactions between ligand and receptor or ligand and ligand (Karpusas et al. 1997; Meister et al. 1986; Radhakrishnan et al. 1996). Comparison of the mu-IFN-CSP with the native IFN-CSP shows that there are three structure differences including two random coils turned into alpha helixes and one alpha helix turned into random coil (Fig. 2B). Figure 2D shows the energy curves and total energy values, which indicate that the mu-IFN-CSP were more stable than the native IFN-CSP. Compared to the native IFN-CSP with 0–2.6% solubility (Fig. 6a) (Lu et al. 2014, 2015b), the solubility of mu-IFN-CSP in *E. coli* was 75.2% before induction conditions optimized (Fig. 6b). In vitro anti-HBV activity study in HepG2.2.15 cells showed that the soluble Mu-IFN-CSP has improved inhibition effect on HBV replication compared to the native IFN-CSP. In this, we speculate that these mutations in Mu-IFN-CSP may relate to not only the activity but also the stability and the solubility.

Previous studies have found that the rare codons have the greatest degree of conformity to lowly expressed genes in the *E. coli* cell, and the expression level may significantly be improved by codon optimization (Sharp and Li 1987; Peng et al. 2004; Xu et al. 2006). There are 18 amino acids in the Mu-IFN-CSP encoded by the rare codons of *E. coli*. Among these, R12, R13, R23, R33, R105, R125, R126, R145, R150, R161, R163, and R164 were encoded by AGG, which was the least used codons of *E. coli* (Xu et al. 2006). To eliminate the influence of rare codons especially the least used codons, AGG, on the Mu-IFN-CSP expression in recombinant *E. coli*, the Mu-IFN-CSP gene was codon-optimized according to the preferred codon usage of *E. coli*.

Many studies are devoted to produce functional interferon using DNA recombinant technology since IFNs have been applied as therapeutic medicine. Most of them have reported that the IFNs are expressed as inclusion bodies in recombinant *E. coli* (Neves et al. 2004; Srivastava et al. 2005; Valente et al. 2004). The refolding of misfolding and aggregation IFNs from inclusion bodies usually need extensive optimization, which makes the refolding procedures cumbersome (Middelberg 2002; Vu et al. 2016). Up to now, it is still hard to produce soluble IFNs in recombinant *E. coli*. In our previous study, the IFN-CSP is also expressed as inclusion bodies even the codon optimization strategy and expression conditions optimization strategy have been used (Lu et al. 2015b). Our present study reported for the first time that liver-targeting interferon mu-IFN-CSP can be expressed as soluble form with 75.2% solubility. In further efforts to increase the soluble expression of mu-IFN-CSP in recombinant *E. coli*, the induction conditions for expression were optimized by an orthogonal test. As reported previously, optimization of induction conditions could be an effective strategy to improve the amount of various recombinant products in the soluble form (Liu et al. 2011; Yan et al. 2012). The present study showed that the solubility of mu-IFN-CSP was 75.2% without any induction conditions optimization. After induction conditions optimized, the solubility of mu-IFN-CSP was up to 98.4%. The most optimal condition was at OD_600_ = 0.9, inducing the culture at 34 °C for 6 h with 0.1 mM IPTG, which is consistent with previous reports that low temperature and low IPTG concentration may improve the solubility of target protein (Vu et al. 2016; Xu et al. 2006).

To study the influence of amino acid positions mutations on the function of mu-IFN-CSP, we also compared the liver tissue binding capacity and in vitro anti-HBV activity of the mutant with the native IFN-CSP. The results of in vitro liver tissue binding study shows that both the native protein and the mutant treatment displayed same degree green fluorescence, which indicate the mutation has no influence on the targeting capacity of the mu-IFN-CSP. The in vitro anti-HBV study in HepG2.2.15 cells showed that the soluble Mu-IFN-CSP has improved inhibition effect on HBV replication compared to the native IFN-CSP. These results are consistent with previous reports in which the mutation relates to the activity (Gangemi et al. 1989; Peek et al. 2004).

In summary, we designed a novel mu-IFN-CSP based on the native IFN-CSP using amino acid positions mutant. The structural comparison and molecular dynamic simulation showed that the Mu-IFN-CSP formed three structure changes and were more stable than the native IFN-CSP. After amino acid mutant, codon-optimization and induction conditions optimization, the solubility of target protein was up to 98.4%. Tissue sections binding and in vitro anti-HBV activity assays demonstrated that the soluble Mu-IFN-CSP has comparable liver tissue binding capacity and higher anti-HBV efficacy compared with the native IFN-CSP. The article reports for the first time that liver-targeting interferon Mu-IFN-CSP can be expressed as soluble form, and also contributes to further support its application as liver-targeting anti-HBV medicine.

## Abbreviations

HBV: hepatitis B virus
*E. coli*: *Escherichia coli*
IFN: interferon
MD: molecular dynamic
RMSD: root mean square deviation
SOE-PCR: splicing by overlapping extension polymerase chain reaction
LB: Luria–Bertani
IPTG: isopropylthio-d-galactoside
SDS-PAGE: sodium dodecyl sulfate polyacrylamide gel electrophoresis
S: soluble supernatant
P: precipitation
LPS: lipopolysaccharides
RP-HPLC: reverse phase high-performance liquid chromatography
DAPI: 4′,6-diamidino-2-phenylindole
HBsAg: hepatitis B s antigen
HBeAg: hepatitis B e antigen
ELISA: enzyme-linked immunosorbent assay
FQ-PCR: fluorescent quantification PCR
SEM: standard error of the mean
ANOVA: Analysis of Variance
HSV: herpes simplex virus
BVDV: bovine viral diarrhea virus
VSV: vesicular stomatitis virus
